# Complications of new-onset atrial fibrillation in critically ill COVID-19 patients admitted to the intensive care unit (ICU): a meta-analysis

**DOI:** 10.1186/s12872-024-04086-5

**Published:** 2024-08-05

**Authors:** Tao Zhang, Ping Gui, Bo Wang

**Affiliations:** 1 Department of Cardiothoracic Vascular Surgery, Renmin Hospital, Hubei University of Medicine, Shiyan, Hubei 442000 People’s Republic of China; 2 Department of Pulmonary and Critical Care Medicine Intervention and Function, Renmin Hospital, Hubei University of Medicine, Shiyan, Hubei 442000 People’s Republic of China

**Keywords:** New-onset atrial fibrillation, Intensive care unit, COVID-19 infection, Mortality, Myocardial infarction, Pulmonary embolism, Mechanical ventilation, Acute kidney injury, Renal replacement therapy

## Abstract

**Background:**

COVID-19 infections can result in severe acute respiratory distress syndrome (ARDS) requiring admission to the intensive care unit (ICU). Cardiovascular manifestation or exacerbation of cardiovascular diseases could be another complication. Cardiac arrhythmias including New-Onset Atrial Fibrillation (NOAF), have been observed in hospitalized patients with COVID-19 infections. In this analysis, we aimed to systematically compare the complications associated with NOAF in critically ill COVID-19 patients admitted to the ICU.

**Methods:**

MEDLINE, EMBASE, Web of Science, the Cochrane database, http://www.ClinicalTrials.gov, Google Scholar and Mendeley were searched for relevant publications based on COVID-19 patients with NOAF admitted to the ICU. Complications including in-hospital mortality, ICU mortality, patients requiring mechanical ventilation, acute myocardial infarction, acute kidney injury, renal replacement therapy and pulmonary embolism were assessed. This is a meta-analysis and the analytical tool which was used was the RevMan software version 5.4. Risk ratios (RR) and 95% confidence intervals (CIs) were used to represent the data post analysis.

**Results:**

In critically ill COVID-19 patients with NOAF admitted to the ICU, the risks of ICU mortality (RR: 1.39, 95% CI: 1.07 – 1.80; *P* = 0.01), in-hospital mortality (RR: 1.56, 95% CI: 1.20 – 2.04; *P* = 0.001), patients requiring mechanical ventilation (RR: 1.32, 95% CI: 1.04 – 1.66; *P* = 0.02) were significantly higher when compared to the control group without AF. Acute myocardial infarction (RR: 1.54, 95% CI: 1.31 – 1.81; *P* = 0.00001), the risk for acute kidney injury (RR: 1.31, 95% CI: 1.11 – 1.55; *P *= 0.002) and patients requiring renal replacement therapy (RR: 1.83, 95% CI: 1.60 – 2.09; *P* = 0.00001) were also significantly higher in patients with NOAF.

**Conclusions:**

Critically ill COVID-19 patients with NOAF admitted to the ICU were at significantly higher risks of developing complications and death compared to similar patients without AF.

## Introduction

COVID-19 infection, caused by the Severe Acute Respiratory Syndrome Coronavirus-2 (SARS-Cov-2), first emerged in China in December 2019 and was declared a pandemic by March 2020 [1]. Since then, there have been over 106 million confirmed cases and 2.3 million deaths globally as of February 2021 [2]. This pandemic has resulted in massive economic losses all around the world [3]. Even though COVID-19 infection mainly affects the lungs causing pneumonia [4], resulting in severe acute respiratory distress syndrome (ARDS) requiring admission to the intensive care unit (ICU) [5], it was observed that cardiovascular manifestation and exacerbation of cardiovascular diseases could be a complication of COVID-19 infection [6]. Cardiac arrhythmias including New Onset Atrial Fibrillation (NOAF), have been observed in hospitalized patients with COVID-19 infections [7].

In this analysis, we aimed to systematically compare the complications associated with NOAF in critically ill COVID-19 patients admitted to the Intensive Care Unit (ICU).

## Methods

### Search databases

MEDLINE, EMBASE, Web of Science, the Cochrane database, http://www.ClinicalTrials.gov, Google Scholar and Mendeley were searched for relevant publications based on COVID-19 patients with NOAF admitted to the ICU.

Reference lists of selective publications were also searched for relevant studies.

### Search strategies

The following searched terms were used:

- COVID-19 infection and intensive care unit;
- COVID-19 infection and atrial fibrillation;
- COVID-19 infection and atrial fibrillation and intensive care unit;
- COVID-19 infection and critical care unit and atrial fibrillation;
- COVID-19 infection and new-onset atrial fibrillation;
- COVID-19 infection and new-onset atrial fibrillation and intensive care unit.

Abbreviations such as AF, NOAF, and ICU were also used during this search process.

### Inclusion and exclusion criteria

The inclusion criteria were:

- Studies including randomized trials or observational studies comparing the clinical outcomes in critically ill COVID-19 patients with NOAF admitted in the ICU;
- Studies mentioned in part (a) reporting clinical outcomes in COVID-19 patients with NOAF versus non-NOAF (control group);
- Studies that included dichotomous data;
- Studies that were published in English.

The exclusion criteria included:

- Studies that did not involve patients who were admitted to the ICU;
- Studies that were literature reviews, brief reviews, narrative reviews, meta-analyses and systematic reviews;
- Repetitive studies.

### Data extraction and quality assessment

The authors independently extracted data from the original studies. Extracted data were carefully cross-checked by the authors, and any disagreement that followed was resolved by consensus.

The following data were extracted:

- Authors names;
- Year of publication;
- Number of COVID-19 participants with and without NOAF respectively;
- Methodological features of the studies;
- General features including the type of study;
- Baseline features of the participants;
- The complications which were reported in each original study;
- The number of events associated with each complication subgroup in both the experimental and the control groups.

Quality assessment was carried out with reference to the Newcastle Ottawa Scale (NOS) [8]. A grade ranging from A to C was given, denoting low, moderate, and high risk of bias.

### Outcomes reported

The endpoints of this analysis included:

- In-hospital mortality;
- ICU mortality;
- Patients requiring mechanical ventilation;
- Acute myocardial infarction;
- Acute kidney injury;
- Renal replacement therapy;
- Pulmonary embolism.

The outcomes which were reported in the original studies have been listed in Table 1.

**Table 1:** Outcomes reported

Studies	Outcomes reported
**Ergün 2021 ** [9]	Length of ICU stay, length of hospital stay, ICU mortality, Hospital mortality, vasopressor requirement, secondary bacterial infection, acute kidney injury, renal replacement therapy, acute myocardial infarction, cardiac injury, acute pulmonary embolism, CPR, ventilator acquired pneumonia
**Kanthasamy 2021 ** [10]	Acute kidney injury, renal replacement therapy, venous thromboembolism, arterial embolism, length of ICU stay, In-hospital mortality
**Kensara 2023 ** [11]	30-day mortality, in-hospital mortality, Mechanical ventilation duration, ICU length of stay, Hospital length of stay, acute kidney injury, mechanical ventilation
**Randy 2021 ** [12]	Mechanical ventilation, death, length of hospital stay
**Rosenblatt 2022 ** [13]	Mortality, ICU death, non-ICU death, major adverse cardiac events, length of hospitalization, mechanical ventilation, myocardial infarction, cardiac arrest, stroke, deep vein thrombosis/pulmonary embolism, renal replacement therapy
**Zakynthinos 2022 ** [14]	Mortality

*Abbreviations*: *ICU* Intensive care unit, *CPR* Cardiopulmonary resuscitation

### Statistical analysis

This is a meta-analysis and the RevMan software version 5.4 was used to carry out this analysis. Heterogeneity [15] was assessed by the Q statistic test as well as the I^2^ statistic test. A subgroup analysis with a *P* value greater than 0.05 was considered statistically insignificant whereas a subgroup analysis with a *P* value less or equal to 0.05 was considered statistically significant.

For the I^2^ statistic test, an I^2^ value less than 50% denoted low heterogeneity, whereby a fixed effect statistical model was used during analysis, whereas an I^2^ value above 50% denoted higher heterogeneity and required the use of a random effect statistical model.

Risk ratios (RR) with 95% confidence intervals (CIs) were used to represent the data following analysis.

In addition, sensitivity analysis was also carried out to ensure that the final results were not influenced by any particular study. Also, publication bias was assessed by visually observing the funnel plot retrieved through the RevMan software.

### Ethical approval

For this meta-analysis, ethical approval was not applicable. Data were extracted from previously published original studies. Therefore, the authors were not involved in any experiment carried out on animals or humans.

## Results

### Search outcomes

The Preferred Reporting Items in Systematic Reviews and Meta-analyses (PRISMA) guideline was followed [16]. Our search resulted in a total number of 268 publications. Following a careful assessment of the titles and abstracts, an initial phase of elimination was carried out, and publications that were irrelevant to this analysis were directly eliminated. Seventy-eight (78) full-text articles were assessed for eligibility.

After carefully assessing the full-text articles, further eliminations were carried out based on the following reasons:

- Literature reviews, brief reviews, narrative reviews, systematic reviews, and meta-analyses (14);
- A control group was not present (6);
- Dichotomous data was absent (2);
- Complications were not reported (6);
- Repetitive studies (44).

**Fig. 1:**
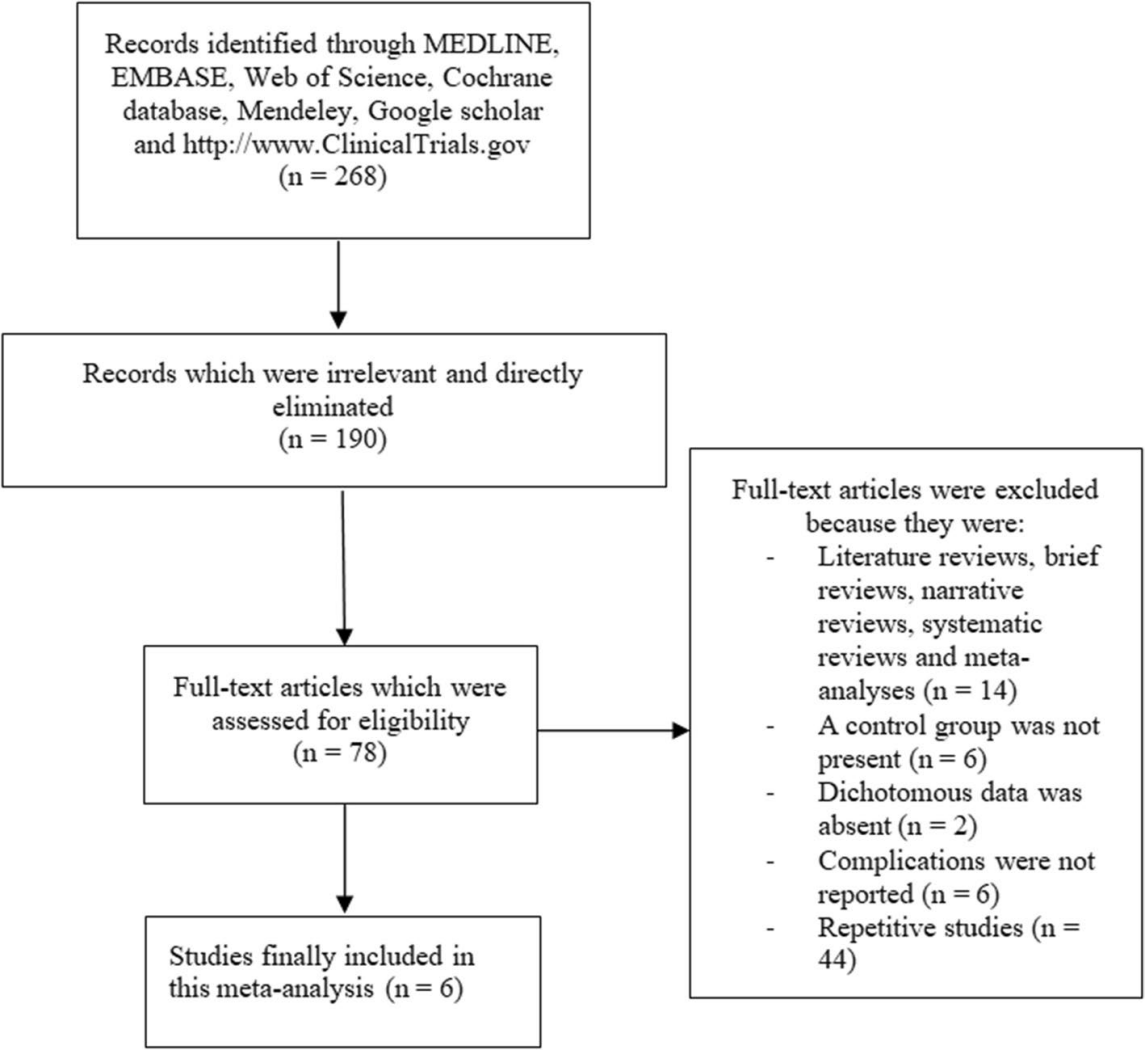
Flow diagram representing the selection of studies for this analysis

Finally, only 6 studies were selected for this analysis. The flow diagram for the study selection has been demonstrated in Fig. 1.

### General features of the studies

A total number of 9213 participants with COVID-19 were included in this analysis, whereby 1184 participants were patients with NOAF admitted to the ICU, whereas 8029 participants were COVID-19 patients without NOAF, as shown in Table 2. The participants’ enrollment period varied from year 2020 to 2021.

**Table 2:** General features of the studies

Studies	Type of study	Number of COVID-19 participants with NOAF in ICU (n)	Number of COVID-19 participants without NOAF in ICU (n)	Participants’ enrollment period	Bias risk score
**Ergün 2021 ** [9]	Retrospective study	37	211	2020 – 2021	B
**Kanthasamy 2021 ** [10]	Retrospective study	16	93	2020	B
**Kensara 2023 ** [11]	Multi-center cohort study	100	300	2020 – 2021	B
**Randy 2021 ** [12]	Multi-center study	34	111	2020	B
**Rosenblatt 2022 ** [13]	Observational study	978	7254	2020 – 2021	B
**Zakynthinos 2022 ** [14]	Prospective study	19	60	2020—2021	B
**Total no of patients (n)**		1184	8029		

*Abbreviations*: *NOAF* New-onset atrial fibrillation, *ICU* Intensive care unit

### Baseline features

The baseline features of the participants have been listed in Table 3. The mean age of the participants varied from 58.0 to 79.0 years with a male predominance ranging from 48.0% to 85.0%. Participants with co-morbidities, including hypertension (52.6% to 83.8%), diabetes mellitus (34.7% to 65.0%), and congestive heart failure (2.00% to 33.0%) were also listed in Table 3.

**Table 3:** Baseline features of the participants

Studies	Age (years)	Males (%)	HBP (%)	DM (%)	CHF (%)
	**NOAF/CL**	**NOAF/CL**	**NOAF/CL**	**NOAF/CL**	**NOAF/CL**
**Ergün 2021 **[9]	79.0/70.0	78.4/69.7	83.8/68.2	37.8/36.5	24.3/14.2
**Kanthasamy 2021 **[10]	65.0/58.0	69.0/85.0	75.0/53.0	63.0/39.0	33.0/2.00
**Kensara 2023 **[11]	73.0/72.0	57.0/56.3	74.0/65.0	63.0/65.0	8.00/9.30
**Randy 2021** [12]	76.0/63.0	57.9/57.9	71.2/71.2	37.4/37.4	-
**Rosenblatt 2022 **[13]	73.0/61.0	60.3/48.0	72.5/56.8	38.2/34.7	17.4/8.50
**Zakynthinos 2022 **[14]	69.7/68.2	74.0/76.0	52.6/76.6	-	-

*Abbreviations*: *NOAF* New-onset atrial fibrillation, *CL* Control group, *HBP* High blood pressure, *DM* Diabetes mellitus, *CHF* Congestive heart failure

### The main results of this analysis

**Fig. 2:**
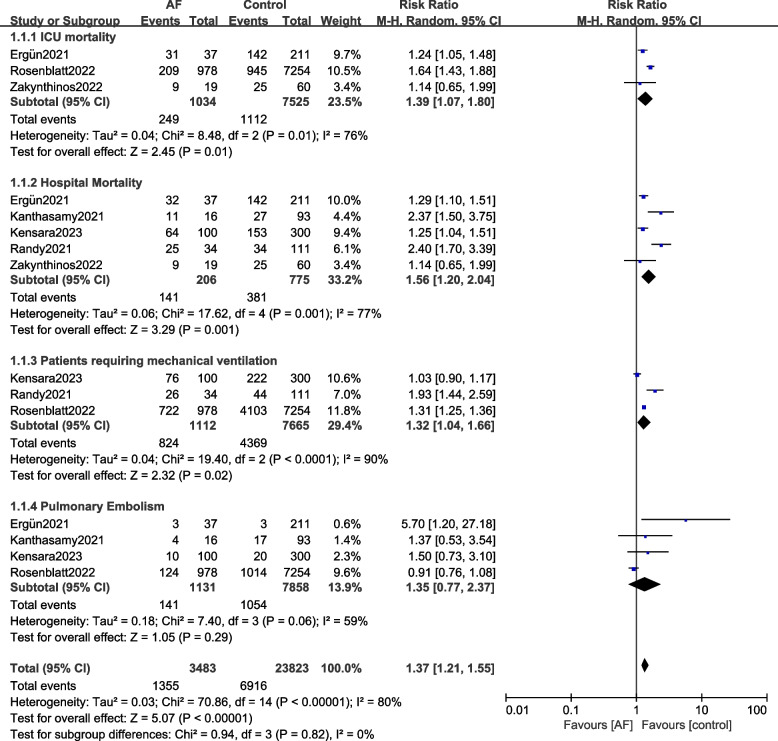
Comparing the complications observed in critically ill COVID-19 patients with versus without new-onset atrial fibrillation admitted to the intensive care unit (A)

**Fig. 3:**
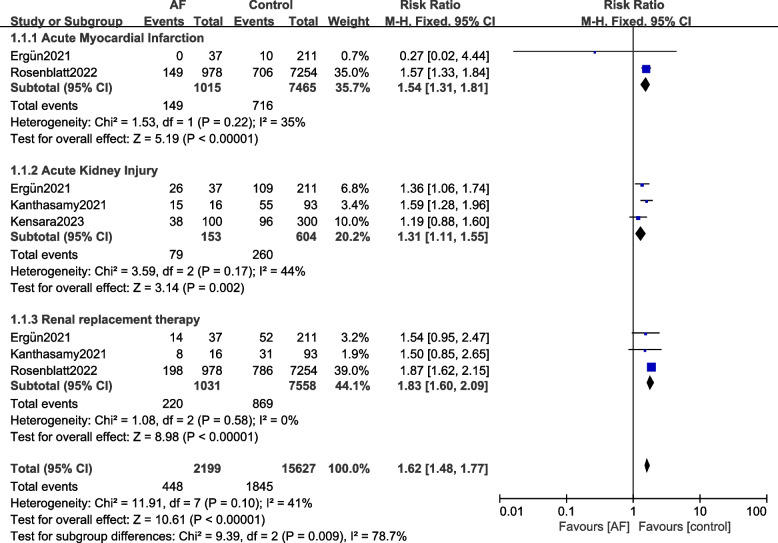
Comparing the complications observed in critically ill COVID-19 patients with versus without new-onset atrial fibrillation admitted to the intensive care unit (B)

Results of this current analysis showed that in critically ill COVID-19 patients with NOAF admitted to the ICU, the risks of ICU mortality (RR: 1.39, 95% CI: 1.07 – 1.80; *P* = 0.01), in-hospital mortality (RR: 1.56, 95% CI: 1.20 – 2.04; *P* = 0.001), patients requiring mechanical ventilation (RR: 1.32, 95% CI: 1.04 – 1.66; *P* = 0.02) were significantly higher when compared to the control group without AF as shown in Fig. 2. Acute myocardial infarction (RR: 1.54, 95% CI: 1.31 – 1.81; *P* = 0.00001), the risk for acute kidney injury (RR: 1.31, 95% CI: 1.11 – 1.55; *P* = 0.002) and patients requiring renal replacement therapy (RR: 1.83, 95% CI: 1.60 – 2.09; *P* = 0.00001) were also significantly higher in patients with NOAF compared to the control group as shown in Fig. 3. However, the risk of pulmonary embolism (RR: 1.35, 95% CI: 0.77 – 2.37; *P* = 0.29) was not significant (Fig. 2).

The main results of this analysis have been tabulated (Table 4).

**Table 4:** Results of this analysis

Outcomes	RR with 95% CI	*P* value	I^2^ value (%)
**In hospital mortality**	1.56 [1.20 – 2.04]	0.001	77
**ICU mortality**	1.39 [1.07 – 1.80]	0.01	76
**Patients on mechanical ventilation**	1.32 [1.04 – 1.66]	0.02	90
**Acute kidney injury**	1.31 [1.11 – 1.55]	0.002	44
**Renal replacement therapy**	1.83 [1.60 – 2.09]	0.00001	0
**Acute myocardial infarction**	1.54 [1.31 – 1.81]	0.00001	35
**Pulmonary embolism**	1.35 [0.77 – 2.37]	0.29	59

*Abbreviations*: *RR* Risk ratios, *CI* Confidence intervals, *ICU* Intensive care unit

**Fig. 4:**
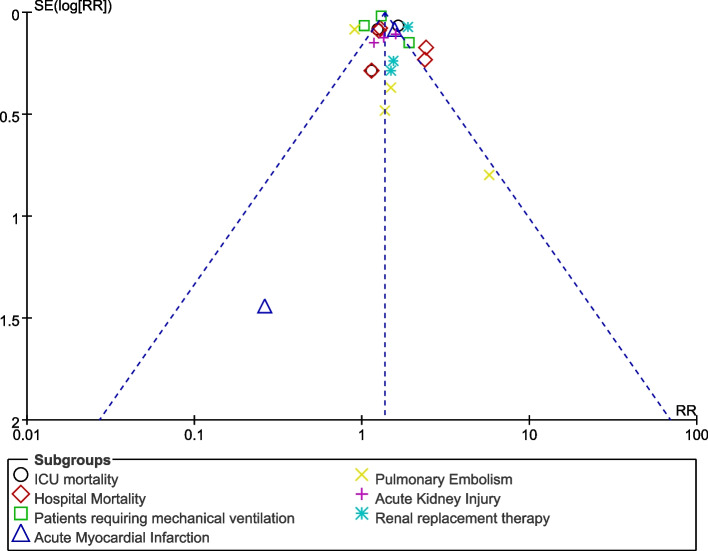
Funnel plot representing publication bias

Consistent results were obtained throughout during sensitivity analysis. Publication bias was visually assessed through the funnel plot. This visual assessment of the funnel plot showed low to moderate evidence of publication bias across the studies that were included during this meta-analysis. The funnel plot is illustrated in Fig. 4.

## Discussion

This current analysis showed that complications, including mortality, acute myocardial infarction, patients requiring mechanical ventilation, acute kidney injury, and patients requiring renal replacement therapy, were significantly higher in COVID-19 patients with NOAF admitted to the ICU.

Several potential reasons have been given for this significantly higher risk of complications in COVID-19 patients with NOAF. A probable mechanism that could contribute to the development of AF in such critical patients could be myocardial damage which might have resulted from viral-induced cardiac injury, giving rise to peri-myocarditis, hypoxemia and acid–base disturbances. The common use of adrenergic drugs in such critical patients could also lead to higher risk of complications [17].

Another causative factor could be secondary bacterial infections which could result in the development of NOAF in such COVID-19 patients. In a study published in the Journal of Infective Public Health [14] based on secondary bacterial infections as a leading factor triggering NOAF in intubated ICU COVID-19 ARDS patients, 24% of the participants were present with NOAF, among which, 89.5% suffered a septic secondary infection showing secondary infection to be a major contributor for the occurrence of NOAF in such COVID-19 patients.

AF was more likely to occur in patients with several co-morbidities including diabetes mellitus, heart failure, coronary artery disease, valvular heart diseases, chronic kidney disease, stroke and obese patients [18]. In addition, NOAF was considered as an independent prognostic indicator for mortality in critically ill COVID-19 patients [19]. Furthermore, a low prevalence of AF was observed in clinically stable COVID-19 patients implying higher risk of NOAF in patients with severe COVID-19 infections [20].

A systematic review and meta-analysis aimed to show the prevalence and impact of AF in hospitalized patients with COVID-19 [21]. The analysis which included a total number of 187,716 COVID-19 patients showed that AF was found in at least 8% of the patients and those COVID-19 patients with AF were older, were more hypertensive and were in a more critical state. It was also stated that AF was associated with an increased risk of mortality in patients with COVID-19 infections. The occurrence of NOAF further contributed to co-morbidities and complications [17].

Similar to our analysis, a study demonstrated atrial arrhythmia-related outcomes in critically ill COVID-19 patients [22]. The study included 200 participants with COVID-19 infections. The authors concluded that atrial tachyarrhythmia was associated with an increased risk of mortality especially in those patients on mechanical ventilation. The authors also stated that if this matter is further explored, it would be beneficial in treating similar patients with COVID-19 infection.

A systematic review showed the risk factors for NOAF in patients admitted to the ICU [23]. The authors suggested age, male gender, pre-existing cardiovascular disease, acute kidney injuries, and acute respiratory failure contribute to the manifestation of NOAF in COVID-19 patients in the ICU.

Finally, in a multi-centre historical cohort study in the Netherlands including patients from 8 hospitals with 3064 hospitalized COVID-19 patients [24], it was shown that NOAF was associated with a higher incidence of mortality and other complications with lesser chance that the patients would be discharged home. This pattern was more prevalent in participants with NOAF compared to those who had AF prior to COVID-19 and the authors concluded that NOAF could be considered a marker for more severe disease. Similarly, a single-center retrospective cohort study including a total number of 492 participants showed NOAF to be considered a risk factor for worse outcomes in ICU patients admitted with severe COVID-19 infections [25].

## Limitations

This study also has limitations. Data were extracted mostly from observational studies and the total number of participants was limited in comparison to other meta-analysis. Even though with such heterogeneous groups, we have been able to reach robust results. Also, several other complications were reported in the original studies but we could not include them in this current analysis since these complications were not reported in several of the studies. In addition, the other co-morbidities were ignored when carrying out this analysis. Furthermore, the COVID-19 treatment modalities and protocols varied in different countries and different hospitals. Therefore, this could also have an impact on the final outcomes.

## Conclusions

Critically ill COVID-19 patients with NOAF admitted to the ICU were at significantly higher risks of developing complications and death compared to similar patients without AF.

## Data Availability

Data which have been used in this study can freely be accessed and are included in the original published articles. References of the original papers involving the data source which have been used in this paper have been listed in the main text of this current manuscript. All data are publicly available in electronic databases.

## Abbreviations

AF: Atrial fibrillation
NOAF: New-onset atrial fibrillation
ICU: Intensive care unit
PE: Pulmonary embolism
AMI: Acute myocardial infarction
AKI: Acute kidney injury
